# Systolic blood pressure and short-term mortality in the emergency department and prehospital setting: a hospital-based cohort study

**DOI:** 10.1186/s13054-015-0884-y

**Published:** 2015-04-09

**Authors:** Anders Kasper Bruun Kristensen, Jon Gitz Holler, Søren Mikkelsen, Jesper Hallas, Annmarie Lassen

**Affiliations:** Department of Emergency Medicine, Odense University Hospital, Sdr Boulevard 29, 5000 Odense C, Denmark; Department of Anesthesiology and Intensive Care Medicine, Odense University Hospital, Sdr Boulevard 29, 5000 Odense C, Denmark; Clinical Pharmacology, Institute of Public Health, University of Southern Denmark, Winslowparken 19, 5000 Odense C, Denmark

## Abstract

**Introduction:**

Systolic blood pressure is a widely used tool to assess circulatory function in acutely ill patients. The systolic blood pressure limit where a given patient should be considered hypotensive is the subject of debate and recent studies have advocated higher systolic blood pressure thresholds than the traditional 90 mmHg. The aim of this study was to identify the best performing systolic blood pressure thresholds with regards to predicting 7-day mortality and to evaluate the applicability of these in the emergency department as well as in the prehospital setting.

**Methods:**

A retrospective, hospital-based cohort study was performed at Odense University Hospital that included all adult patients in the emergency department between 1995 and 2011, all patients transported to the emergency department in ambulances in the period 2012 to 2013, and all patients serviced by the physician-staffed mobile emergency care unit (MECU) in Odense between 2007 and 2013. We used the first recorded systolic blood pressure and the main outcome was 7-day mortality. Best performing thresholds were identified with methods based on receiver operating characteristics (ROC) and multivariate regression. The performance of systolic blood pressure thresholds was evaluated with standard summary statistics for diagnostic tests.

**Results:**

Seven-day mortality rates varied from 1.8 % (95 % CI (1.7, 1.9)) of 112,727 patients in the emergency department to 2.2 % (95 % CI (2.0, 2.5)) of 15,862 patients in the ambulance and 5.7 % (95 % CI (5.3, 6.2)) of 12,270 patients in the mobile emergency care units. Best performing thresholds ranged from 95 to 119 mmHg in the emergency department, 103 to 120 mmHg in the ambulance, and 101 to 115 mmHg in the MECU but area under the ROC curve indicated poor overall discriminatory performance of SBP thresholds in all cohorts.

**Conclusions:**

Systolic blood pressure alone is not sufficient to identify patients at risk regardless of the defined threshold for hypotension. If, however, a threshold is to be defined, a systolic blood pressure threshold of 100 to 110 mmHg is probably more relevant than the traditional 90 mmHg.

**Electronic supplementary material:**

The online version of this article (doi:10.1186/s13054-015-0884-y) contains supplementary material, which is available to authorized users.

## Introduction

Systolic blood pressure (SBP) measurements are widely used in clinical practice to assess the circulatory state of acutely ill patients. Arterial hypotension has traditionally been defined as a SBP below 90 mmHg [1]. However, this threshold seems to have prevailed more because of traditions and less because of scientific merit as no evidence supporting a threshold of 90 mmHg seems to exist.

Recent studies have supported a redefinition of arterial hypotension. Short-term mortality has been shown to increase at SBP levels below 110 mmHg for a population of trauma patients [2] and below 100 mmHg in a cohort of patients in the prehospital setting [3]. These limits remain to be established in a broad population of patients presenting to the emergency department (ED). Applying the traditional SBP threshold of 90 mmHg probably results in a considerable number of patients with increased risk of death being overlooked. These patients might benefit from an increased intensity of observation and care. The disadvantage of using a higher threshold is that too many patients with low mortality risk may be put through extensive surveillance or therapy without benefit.

We aimed to define SBP thresholds indicating increased risk of 7-day mortality in the ED, ambulances, and physician-staffed mobile emergency care units (MECU) in the region of Southern Denmark and to evaluate the prognostic value of these SBP thresholds in the above settings hypothesizing that the traditional SBP threshold of 90 mmHg performed poorly in identifying patients at risk and that a more relevant SBP threshold could be identified.

## Methods

### Study design and setting

This was a retrospective, hospital-based cohort study of all adult patients arriving at the ED of Odense University Hospital in the period November 1995 to December 2011, all adult patients in ambulances arriving at the ED of Odense University Hospital in the period January 2012 to October 2013, and all adult patients serviced by the MECU operating in the area around Odense in the period October 2007 to December 2013.

Odense University Hospital is a 1,100-bed university teaching hospital with a mixed rural-urban contingency population of 290,000. Medical and surgical specialties are represented at the ED that acts as a general as well as a Level 1 trauma center with a total of approximately 48,000 contacts per year. A dispatch center receives all health-related emergency calls in the region of Southern Denmark and decides on the appropriate response using a nationwide dispatch protocol [4,5]. Depending on the emergency level, an ambulance will be directed to the scene and, based on the degree of severity of the situation, a MECU may be dispatched as well.

In Denmark, prehospital and hospital health services are provided free of charge to all citizens as part of the tax-funded healthcare system.

### Participants

We included all patients 18 years of age or above who were alive at presentation to the ED/ambulance, who had SBP measured and recorded at presentation, and who had a valid Danish personal identification number [6]. If a patient had multiple encounters over the study period, only the first was included within each cohort.

### Variables and outcomes

The primary outcome variable was 7-day mortality while 30-day mortality was included as a secondary outcome. The primary exposure variable was the first recorded SBP value at presentation. Blood pressures were measured non-invasively with a manual cuff and sphygmomanometer or with an automated oscillometric device.

At individual level, we included information on the additional variables: age, gender, time of contact (07:00 to 14:59, 15:00 to 22:59, 23:00 to 06:59), rural/urban residence [7], Charlson Comorbidity Index (0, 1 to 2, >2) [8], and essential hypertension as defined by a discharge diagnosis of the International Classification of Diseases (ICD)-10 codes I10* or ICD-8 codes 401* within 10 years from the index date or a redeemed prescription of antihypertensive medication (ATC codes: C02*, C03*, C07*, C08*, C09*) within 90 days before the index date.

### Data sources and measurement

#### Database

All patients presenting to the ED, except those with minor orthopedic complaints, had vital signs measured by a nurse at arrival as part of the routine procedure. We extracted SBP values by means of a self-written text mining algorithm based on regular expressions, a method which has proved valuable for extraction of numerical variables in other settings [9] and manually validated in 500 files for this cohort with a sensitivity of 95.8% (95% confidence interval (CI) (91.2, 98.5)) and a specificity of 100% (95% CI (99.0, 100)). Data on patients treated by ambulances alone were recorded on paper by the attending emergency medical technicians. These data were subsequently stored in a research database. Following each MECU run, patient characteristics including tentative diagnosis, vital signs, and administered treatment, were entered into the MECU database. The study periods of the three cohorts reflected the period for which data was available.

#### Population-based registers

Using the unique Danish personal identification number, we retrieved and linked supplemental information from several large population-based registers [6]. Data on previous discharge diagnoses was extracted from the Danish National Patient Register [10]. We used all discharge diagnoses within the last 10 years to construct the Charlson Comorbidity Index [11]. Data on time of birth, vital status, migration status, and municipality of residence was extracted from the The Danish Civil Registration System [6]. Rural/urban residence was defined on basis of municipality of residence on the index date [7]. Data on previously redeemed prescriptions was extracted from Odense Pharmacoepidemiological Database [12,13].

#### Analysis

Basic characteristics were described using, as appropriate, numbers and percentages, means and standard deviations, or medians, 25^th^ percentiles, and 75^th^ percentiles. For comparisons between the three cohorts we used the chi-square test for categorical variables and the Kruskal-Wallis equality-of-populations rank test for continuous variables.

The association between SBP and 7-day mortality was described by means of logistic regression modeling SBP using a restricted cubic spline with 5 knots [14,15]. Goodness of fit was assessed with the Hosmer-Lemeshow chi-square test. As a pragmatic, clinically relevant SBP threshold, we identified the SBP level corresponding to a predefined 7-day mortality rate of 10%.

In order to assess the overall discriminative ability of SBP thresholds in predicting 7-day mortality, we made receiver operating characteristic (ROC) curves and calculated area under the ROC curves (AUROC). Because mortality increased in both ends of the SBP scale and we aimed to identify the lower SBP threshold, we restricted the ROC curve to patients with SBPs below the value where the ROC curve crossed the diagonal reference line. We tested the null hypothesis that AUROCs were equal in the three cohorts (corresponding to equal overall discriminatory ability of SBP thresholds) by means of the chi-square test [16].

The best performing SBP threshold with regard to optimal sensitivity-specificity trade-off was estimated by means of the ROC-based Youden Index [17]. Furthermore, we identified the optimal thresholds by testing each SBP threshold in the range 80 to 120 mmHg against the outcome, a method referred to as the minimum *P* value approach [18]. This was achieved by calculating z-statistics for each dichotomous SBP threshold variable on 7-day mortality in crude as well as adjusted logistic regression analyses. The adjusted analyses included the covariates mentioned under v*ariables and outcomes* and were checked for multicolinearity and plausible interactions. The clinical performance of the following SBP thresholds <90 mmHg, <100 mmHg, <110 mmHg, and <120 mmHg were evaluated using sensitivity, specificity, likelihood ratios and predictive values. The 95% confidence intervals for these test statistics were calculated using exact binomial methods. Statistical analyses were performed using Stata version 13.1 (Stata Corporation LP, College Station, TX, USA).

#### Ethics committee approval

The study was approved by the Danish Data Protection Agency (J No. 2013-41-2580) and patient record access was approved by the Danish National Board of Health (J No. 3-3013-485/1). Approval by an Ethics Committee or informed consent is not required for register-based research in Denmark. The reporting of this study conforms to the Strengthening the Reporting of Observational Studies in Epidemiology (STROBE) statement [19].

## Results

### Participants

**Figure 1:**
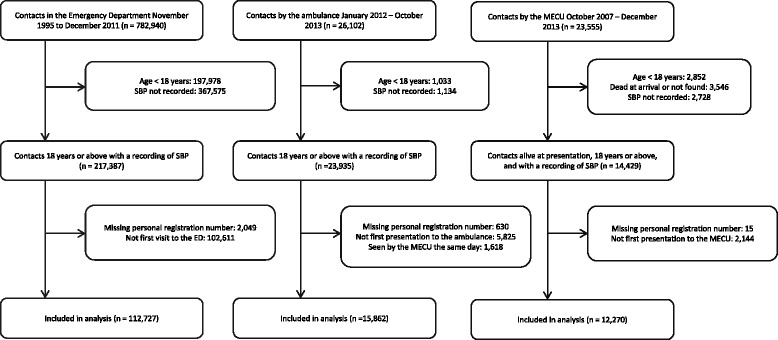
**Recruitment of the three cohorts.**

A total of 112,727 patients were included in the ED cohort, 15,862 in the ambulance cohort, and 12,270 in the MECU cohort (Figure 1). Baseline characteristics differed between the three cohorts with regard to demographic as well as clinical variables. The prehospital population was older and had higher Charlson Comorbidity Indices compared to the ED population (Table 1). Seven-day mortality was 1.8% (95% CI (1.7, 1.9)) in the ED cohort, 2.2% (95% CI (2.0, 2.5)) in the ambulance cohort, and 5.7% (95% CI (5.3, 6.2)) in MECU cohort.

**Table 1:** Baseline characteristics for three emergency populations*

	**Emergency dep.**	**Ambulance**	**MECU**	***P*** **value** ^**†**^
All patients, n	112,727	15,862	12,270	
Male, n (%)	59,523 (53)	7,778 (49)	7,028 (57)	<0.001
Essential hypertension, n (%)	26,592 (24)	7,057 (46)	5,292 (43)	<0.001
Charlson Comorbidity Index, n (%)				<0.001
0	83,068 (74)	8,204 (52)	6,595 (54)	
1 - 2	22,379 (20)	4,949 (31)	3,655 (30)	
>2	7,280 (7)	2,709 (17)	2,020 (17)	
Age, median [25^th^-75^th^ percentile]	49 [30-69]	64 [44-78]	60 [42-74]	<0.001
Systolic blood pressure, mean ± s.d.	148 ± 28	145 ± 28	141 ± 31	<0.001
Diastolic blood pressure, mean ± s.d.	85 ± 16	84 ± 18	84 ± 20	<0.001
Heart rate, mean ± s.d.	84 ± 19	87 ± 20	92 ± 25	<0.001
7-day mortality, n (%)	2,065 (1.8)	356 (2.2)	703 (5.7)	<0.001
30-day mortality, n (%)	3,503 (3.1)	789 (5.0)	1,063 (8.7)	<0.001

^*^Values expressed as total number (fraction), medians [25^th^ percentile to 75^th^ percentile], or mean ± standard deviation (s.d.), as appropriate. ^†^Chi-squared test for categorical variables and Kruskal-Wallis test for continuous variables. MECU, mobile emergency care unit.

### Association between systolic blood pressure and mortality

**Figure 2:**
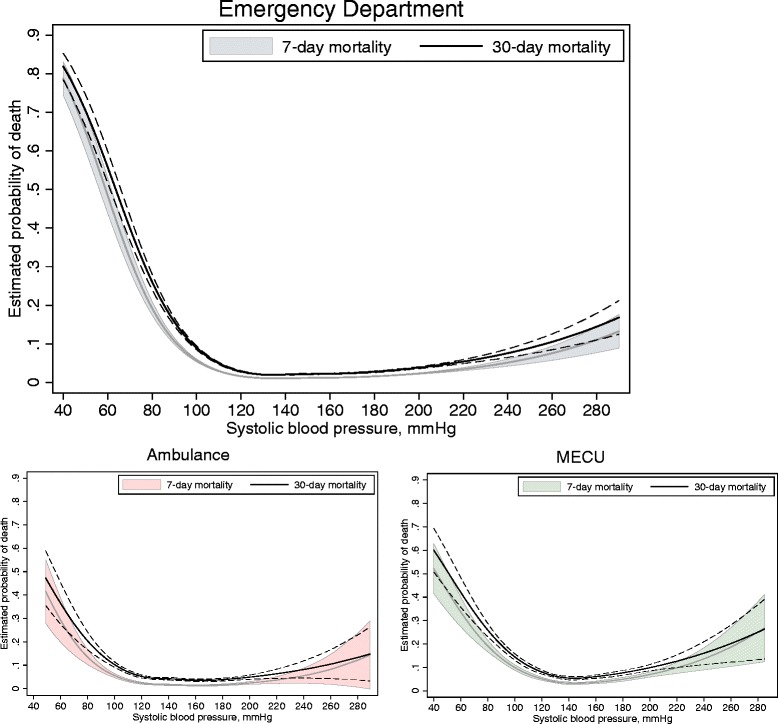
**Seven- and 30-day mortality according to systolic blood pressure.** Grey line corresponds to 7-day mortality, black line to 30-day mortality. 95% CIs are indicated with dashed lines. CI, confidence interval.

The unadjusted logistic regression models revealed a *u*-shaped relationship between SBP and 7-day mortality as well as 30-day mortality. Seven-day mortality began to increase at SBP values around 110 mmHg in the ED and around 120 mmHg in the ambulance and MECU (Figure 2). The estimated probability of death within 7 days reached 10% at SBPs below 92 mmHg (95% CI (90, 93)) for the ED cohort, 87 mmHg (95% CI (81, 91)) for the ambulance cohort, and 100 mmHg (95% CI (97, 104)) for the MECU cohort.

### Prognostic performance of systolic blood pressure thresholds

**Figure 3:**
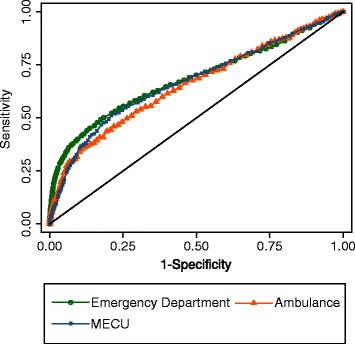
**Receiver operating characteristic curve of systolic blood pressure for 7-day mortality.**

ROC curves for the three cohorts are shown in Figure 3. The AUROC for 7-day mortality was 0.68 (95% CI (0.67, 0.70)) for the ED cohort. For the ambulance cohort the AUROC was 0.65 (95% CI (0.61, 0.69)) while it was 0.67 (95% CI (0.64, 0.70)) for the MECU cohort. We found no evidence of differences in the ability of SBP to discriminate 7-day survivors from nonsurvivors between the three settings, that is, dissimilar AUROCs for the three cohorts (*P* value: 0.30).

### Establishing a threshold

We identified statistically optimal SBP thresholds for predicting 7-day mortality using two different methods, Youden Index and the minimum *P* value approach. The Youden index corresponds to sensitivity + specificity – 1 and was maximal at SBP thresholds of 119 mmHg in the ED, 120 mmHg in the ambulance, and 115 mmHg in the MECU. The multivariate regression based minimum *P* value approach yielded maximal z-statistics at SBP thresholds of 100 mmHg in the ED, 104 mmHg in the ambulance, and 110 mmHg in the MECU while the univariate analyses yielded maximal z-statistics at SBP thresholds of 95 mmHg in the ED, 103 mmHg in the ambulance, and 101 mmHg in the MECU (see Additional file 1).

With the aim of describing the clinical applicability of a range of possible SBP thresholds including the traditional 90 mmHg and the statistically optimal threshold of 110 mmHg, Table 2 summarizes sensitivity, specificity, predictive values, likelihood ratios, as well as the false positive/false negative trade-off for increasing the threshold from 90 mmHg. The negative predictive value was 98% or above for all cut points of SBP in the ED and ambulance whereas it was around 95% in the MECU. In contrast, the positive predictive value was higher in the MECU (11 to 21%) than in the two other cohorts (5 to 22%) for most SBP cut points.

**Table 2:** **Summary statistics of possible systolic blood pressure thresholds in predicting 7-day mortality for the three cohorts**

	**Sensitivity**	**Specificity**	**PPV**	**NPV**	**LR+**	**LR-**	**FP**	**FN**	**FP/FN trade-off** ^*^
Emergency department									
<90 mmHg	12 (11-14)	99 (99-99)	22 (20-25)	98 (98-98)	15 (14-18)	0.89 (0.87-0.9)	871	1815	1 (ref)
<100 mmHg	19 (17-20)	98 (98-98)	15 (14-16)	98 (98-99)	9.4 (8.5-10)	0.83 (0.81-0.85)	2193	1679	10
<110 mmHg	27 (25-29)	94 (94-95)	8 (8-9)	99 (99-99)	4.8 (4.5-5.2)	0.78 (0.76-0.8)	6116	1512	17
<120 mmHg	36 (33-38)	87 (87-87)	5 (5-5)	99 (99-99)	2.7 (2.6-2.9)	0.74 (0.72-0.76)	14329	1331	28
Ambulance									
<90 mmHg	10 (7-13)	99 (98-99)	13 (9-18)	98 (98-98)	6.7 (4.8-9.4)	0.92 (0.88-0.95)	228	321	1 (ref)
<100 mmHg	18 (14-22)	97 (96-97)	11 (8-13)	98 (98-98)	5.2 (4.1-6.5)	0.85 (0.81-0.89)	540	292	11
<110 mmHg	27 (22-32)	92 (92-93)	7 (6-9)	98 (98-98)	3.4 (2.9-4.1)	0.79 (0.74-0.84)	1214	260	16
<120 mmHg	37 (32-43)	84 (83-84)	5 (4-6)	98 (98-99)	2.3 (2-2.6)	0.75 (0.69-0.81)	2540	223	24
MECU									
<90 mmHg	16 (14-19)	96 (96-97)	21 (17-24)	95 (95-95)	4.3 (3.6-5.2)	0.87 (0.84-0.9)	440	588	1 (ref)
<100 mmHg	25 (22-29)	93 (92-93)	18 (16-21)	95 (95-96)	3.6 (3.1-4.2)	0.8 (0.77-0.84)	813	524	6
<110 mmHg	34 (31-38)	88 (87-88)	14 (13-16)	96 (95-96)	2.7 (2.5-3.1)	0.75 (0.71-0.79)	1443	462	8
<120 mmHg	43 (40-47)	79 (78-80)	11 (10-12)	96 (95-96)	2.1 (1.9-2.3)	0.72 (0.67-0.76)	2417	398	10

^*^The FP/FN trade-off expresses the additional number of false positive results for one less false negative result that comes with increasing the systolic blood pressure threshold from 90 mmHg. Values in parentheses are 95% confidence intervals. PPV, positive predictive value; NPV, negative predictive value; LR+, positive likelihood ratio; LR-, negative likelihood ratio; FP, false positives; FN, false negatives; MECU, mobile emergency care unit.

## Discussion

We aimed to define SBP thresholds indicating increased risk of 7-day mortality in the ED and in the prehospital setting and to evaluate the prognostic value of these. The SBP thresholds with highest statistical performance in predicting 7-day mortality were around 110 mmHg. Despite the strong association between SBP and mortality, the performance of SBP in predicting 7-day mortality was poor across all three cohorts and SBP by itself is not an adequate tool for risk stratification in the ED or in the prehospital population.

We initially investigated the crude association between 7-day mortality and systolic blood pressure. Mortality began to increase at SBPs around 110 to 120 mmHg and was greatly increased at 90 mmHg. These findings are consistent with previous studies on acutely ill medical patients in the prehospital setting [3] and on trauma patients in the ED and in the prehospital setting [2,20-22]. The fact that mortality begins to increase at SBPs above 90 mmHg has been used as an argument against the traditional threshold of 90 mmHg for primary identification of patients with severely impaired circulatory status. Our results indicate that there might be a need for extra careful patient management at SBPs up to 100 to 110 mmHg at presentation to the ambulance or at arrival to the ED.

In the clinical setting, the definition of an optimal SBP threshold depends on the relative importance of a false negative result versus a false positive result. For the ED cohort, increasing the threshold from 90 mmHg to 110 mmHg would result in an increase in the number of false positives from 871 to 6,116 patients. On the other hand, we would detect (and possibly prevent) 303 additional patients who died within 7 days. Changing the threshold for hypotension from 90 mmHg to 110 mmHg thus represents a trade-off with 17 additional false positive results for 1 less false negative result. For the ambulance this trade-off is 16:1 and for the MECU it is 8:1. It should be noted that these trade-offs depend on the pretest probabilities, which, in this case, equal the overall 7-day mortality rate. The differing mortality rates partly explain these dissimilarities in trade-off.

If we consider a false negative result more serious than a false positive result, this would speak in favor of higher SBP thresholds. A scenario where there is a low cost of falsely classifying a low-risk patient as a high-risk patient would involve implementation of low-risk, low-cost interventions, for example arterial blood gas analysis and lactate measurement for further evaluation of the hemodynamic state of the patient. If the intervention was more costly, for example medical emergency team activation, applying a threshold in the higher range would not be suitable as this would lead to a substantial increase in false positives and allocation of limited resources from patients in need to patients at low risk of death.

The above considerations are especially relevant from an administrative point of view. When the physician faces his patient, the interesting question is whether, given a certain SBP value, this patient is at increased risk of death or an adverse outcome. The positive likelihood ratio is a simple and helpful parameter for this task (for example a patient presenting to the ED who dies within 7 days is 16 times as likely to have a SBP below 90 mmHg than a patient who survives). However, as the negative likelihood ratios range between 0.72 and 0.92, 7-day mortality cannot with confidence be ruled out on the basis of any of the suggested SBP thresholds. Thus, SBP thresholds alone cannot, regardless of whether the threshold is low or high, rule out risk of death. The weak overall performance of SBP in predicting 7-day mortality might be explained by the fact that acute circulatory failure can include but is not limited to a low SBP. In parallel, a recent consensus conference report omitted hypotension from the definition of shock [23]. It is possible that a combination of SBP, heart rate (HR) and lactate as a simple combined indicator of early circulatory failure can prove a high clinical performance but this still remains to be tested.

### Study strengths and limitations

We used three large hospital-based cohorts of acutely ill unselected patients treated in three different clinical settings. Due to the unique personal registration numbers in Denmark, we had no loss of follow-up and were able to identify all included patients in the population-based registers. Data on vital signs was collected prospectively as part of the daily routine documentation for all patients in the prehospital setting and for ED patients where it was deemed relevant by the triaging nurse. Aiming to avoid bias from repeated measurements, we included only the first contact if several encounters were registered for each patient.

As this was a retrospective study using three large datasets, some limitations apply. The ED cohort consisted of all adult newcomers who had SBP measured at arrival. Patients who did not have SBP measured at arrival at the ED were not included in the study (n = 367,575). These patients suffered from minor complaints where the triaging nurse did not deem a SBP measurement relevant. This must be kept in mind when interpreting our findings from the ED. The relevant population is thus patients with medical complaints and trauma severe enough to warrant a SBP measurement. The accuracy of SBP measurements by auscultation and automatic oscillometric devices might be low, especially in the prehospital setting [24,25]. If substantial, the inaccuracy of SBP measurements would bias the association between SBP and mortality as well as the prognostic performance of SBP thresholds toward lower values. In the majority of cases, SBP was measured using automatic devices but the method of measurement was not known on an individual basis.

Another limitation is that we do not know the potential added value of using higher SBP thresholds to the management of patients. Experienced nurses and physicians can accurately predict the mortality of acute patients by global assessment alone [26]. Whether an elevated SBP threshold might improve their assessment and management is currently unknown.

We used the first recorded SBP value. This is a ‘snapshot’ of the circulatory state of the patient and does not reflect change over time. It is, however, still the principal measure of blood pressure levels in emergency research and in clinically applied triage models and clinical prediction rules.

The populations in the three cohorts were from the same geographical area. This might hamper the external validity of our study. However, the three cohorts differed significantly with regards to demographic and clinical characteristics, and our results are comparable to those of a recent study on prehospital encounters in Washington, USA [3].

We were not able to distinguish between trauma and nontrauma cases. An average of one to two major trauma cases was received per day in the study period.

## Conclusions

Despite the strong association between SBP and short-term mortality, SBP thresholds will not by themselves be sufficient for risk stratification regardless of where the threshold is placed. If a threshold is to be defined, a SBP of 100 to 110 mmHg is probably a more relevant threshold than the traditional 90 mmHg.

## Key messages

- Seven-day mortality increases at systolic blood pressures below 110 mmHg in the ED and 120 mmHg in the prehospital setting.
- Although there is a strong association between systolic blood pressure and 7-day mortality, systolic blood pressure does not perform sufficiently well as a single predictor of short-term mortality.
- If systolic blood pressure thresholds are to be defined, a threshold of 100–110 mmHg is probably more relevant than the traditional threshold of 90 mmHg.

## Additional file

Additional file 1:
**Z-statistics from crude and adjusted logistic regression models of dichotomized systolic blood pressure thresholds in the range 80 to 120 mmHg and 7-day mortality.**

## Abbreviations

AUROC: Area under receiver operating characteristic curve
CI: Confidence interval
ED: Emergency department
HR: Heart rate
ICD: International classification of diseases
MECU: Mobile emergency care unit
ROC: Receiver operating characteristic
SBP: Systolic blood pressure
STROBE: Strengthening the reporting of observational studies in epidemiology
