# Supplementary figures and images for: Systolic blood pressure and short-term mortality in the emergency department and prehospital setting: a hospital-based cohort study
Source: Crit Care. 2015 Apr 9;19(1):158. doi: 10.1186/s13054-015-0884-y (PMC4412041; doi:10.1186/s13054-015-0884-y)

# Emergency Department

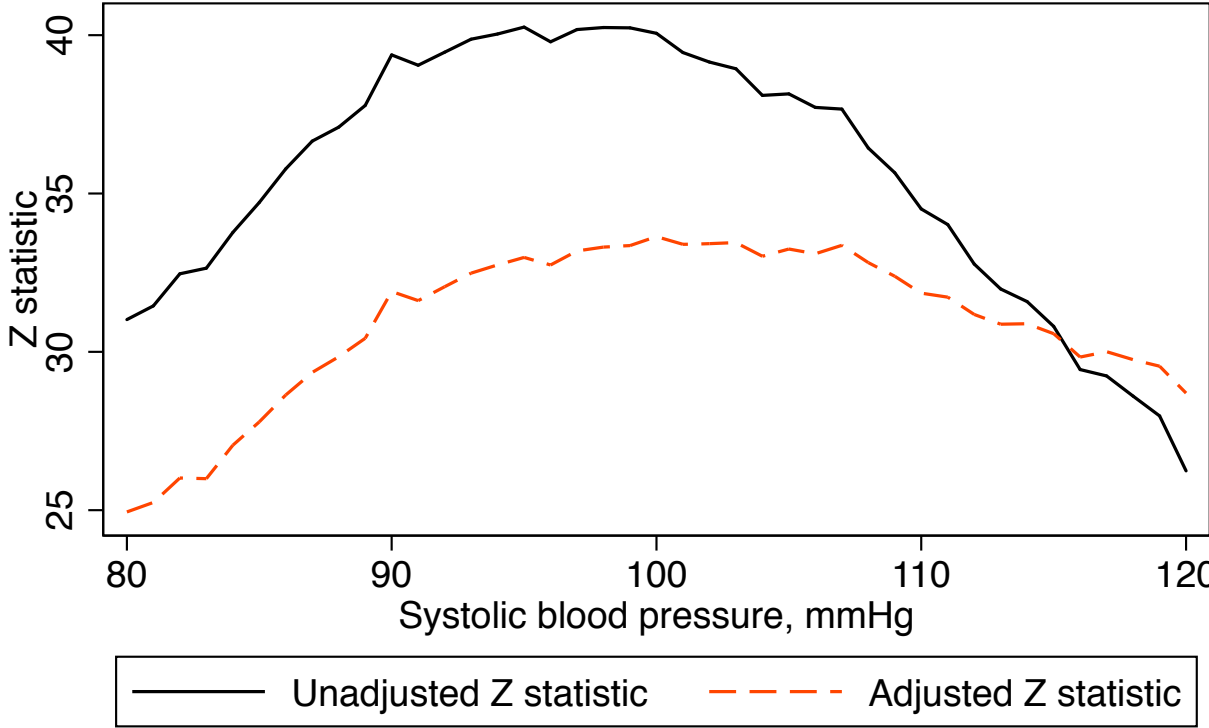

# Ambulance

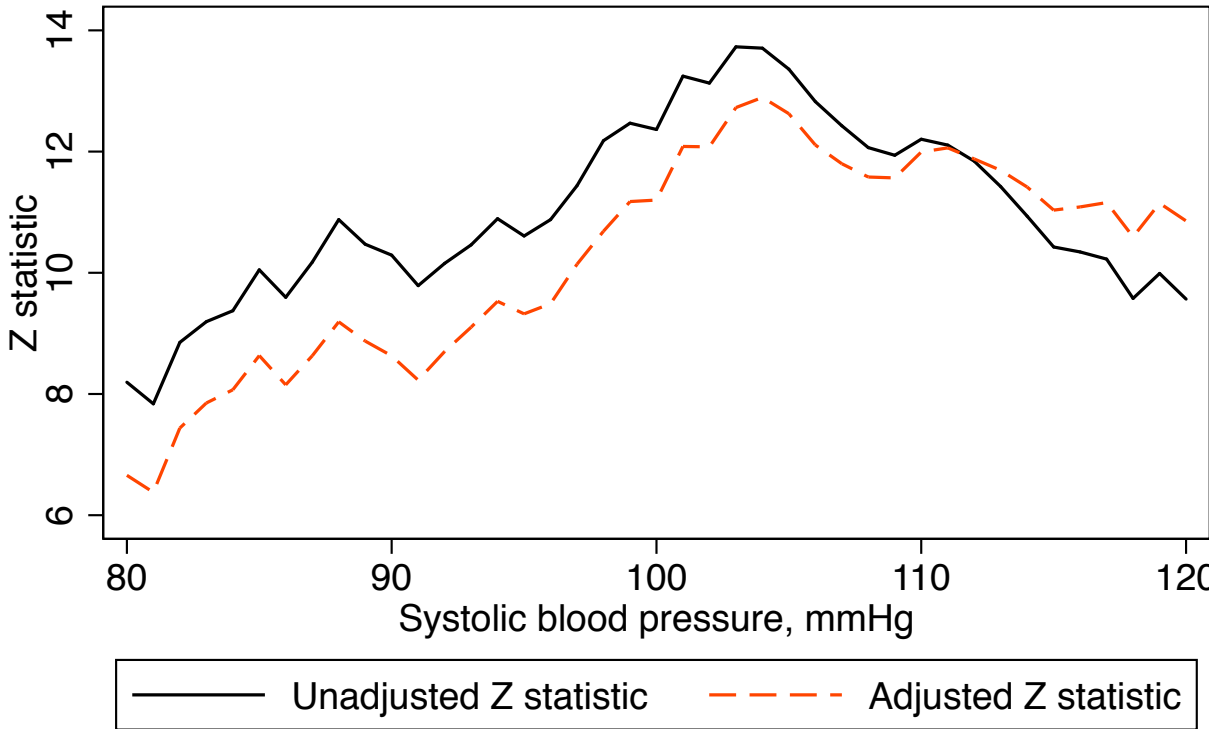

# MECU

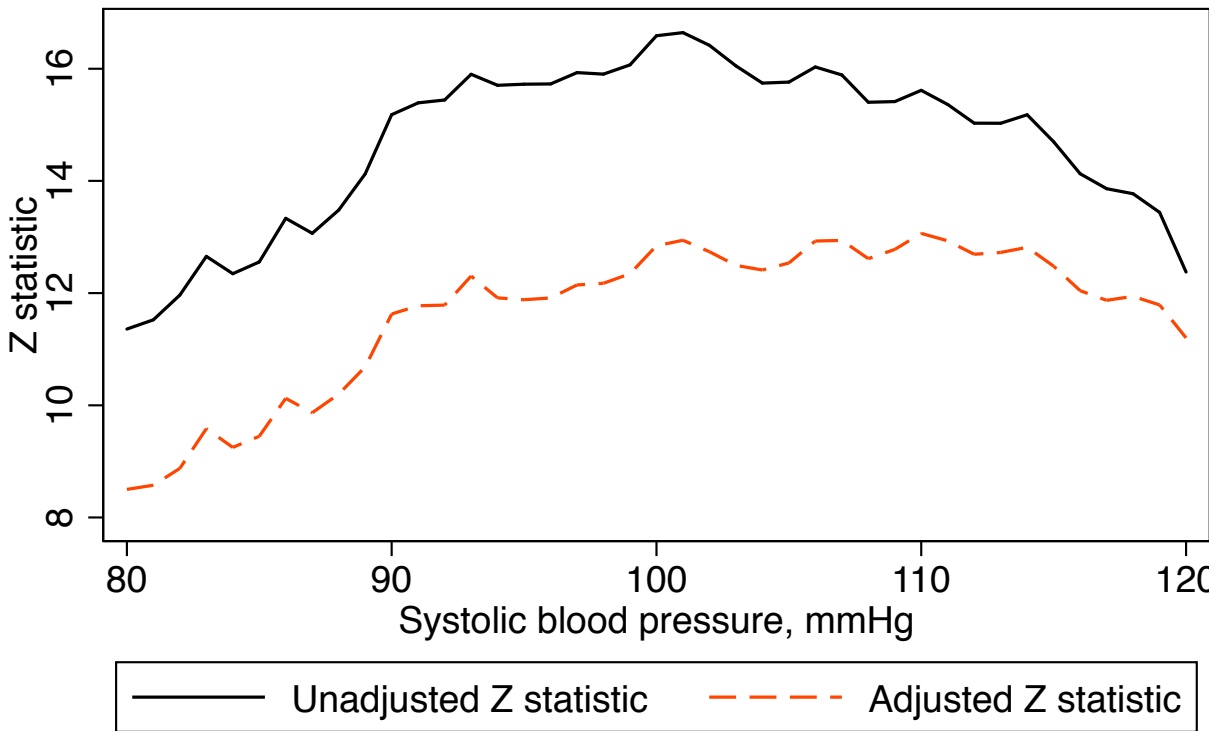

Supplement: Additional file 1: — Z-statistics from crude and adjusted logistic regression models of dichotomized systolic blood pressure thresholds in the range 80 to 120 mmHg and 7-day mortality. [file 13054_2015_884_MOESM1_ESM.pdf]
